# Clinical outcomes after endovascular thrombectomy in different triage methods

**DOI:** 10.1016/j.heliyon.2023.e19113

**Published:** 2023-08-14

**Authors:** Fucheng Jiang, Wenpeng Yin, Jianwen Jia, Hongliang Zhong, Hongchao Yang, Jvmei Huang, Yang Wang, Yunpeng Liu, He Liu

**Affiliations:** a Department of Neurosurgery, Beijing Chaoyang Hospital, Capital Medical University, Beijing, China; b Department of Interventional Radiology and Vascular Surgery, Peking University International Hospital, Beijing, China; c Department of Emergency, Beijing Chaoyang Hospital, Capital Medical University, Beijing, China

**Keywords:** Drip and ship, Mothership, Acute ischemic stroke, Bridging thrombolysis

## Abstract

**Objective:**

The purpose of this study was to evaluate the effectiveness and safety of drip and ship (DS) for acute ischemic stroke (AIS) by comparing three treatment strategies: 1) patients seen at a primary stroke center, started on emergency intravenous thrombolysis and then transported to a comprehensive stroke center (drip and ship, DS); 2) patients immediately transferred to comprehensive stroke center without starting intravenous thrombolysis, for mechanical thrombectomy (non-drip and ship, non-DS); and 3) patients admitted directly to the comprehensive stroke center for assessment and subsequent bridging thrombolysis (mothership, MS).

**Methods:**

We retrospectively reviewed the data of patients that underwent mechanical thrombectomy for AIS from November 2020 to May 2022 at our institution. Patients were divided into three groups: DS, non-DS, and MS. Time course, multimodal CT features and clinical results were compared among the three groups.

**Results:**

The study included 62 patients, with 19, 18, and 25 patients in DS, non-DS, and MS groups, respectively. Baseline characteristics did not differ among the three groups. The DS group had a significantly longer median onset to groin time than the MS group (395 min vs 244 min; *P* < 0.001), a significantly shorter onset to primary stroke center time than the non-DS group (90 min vs 463 min; *P* < 0.001), and a longer primary stroke center to groin puncture time than the non-DS group (277 min vs 162 min; *P* = 0.002). The onset to needle time was longer in the MS group than the DS group (151.2 min vs 111.8 min; *P* = 0.041). The intravenous thrombolysis to puncture time was shorter in the MS group compared with DS (56 min vs 278 min; *P* < 0.001). No significant differences were present among groups in post-operative variables measured.

**Conclusions:**

DS is a safe and effective method, with no increased risk of postoperative complications or death compared to non-DS and MS methods. The study provides a reference for the selection of transport modes for AIS patients.

## 1 Introduction

For patients with acute ischemic stroke (AIS), early cerebral reperfusion is key to successful treatment. Emergency intravenous thrombolysis (IVT) within 4.5 h of symptom onset is recommended in guidelines and has been shown to be safe and effective. However, the efficacy of IVT in patients with large vessel occlusion (LVO) is limited [1]. In recent years, with the advancement of materials and concepts, mechanical thrombectomy (MT) has gradually become a meaningful method of cerebral reperfusion in patients with LVO. In 2018, the time window for MT was broadened after the DAWN and DEFUSE-3 trials were published, greatly increasing the potential number of patients that could benefit from MT [2,3]. China is a country with vast territory, but only 466 comprehensive stroke centers (CSCs) are available for thrombectomy [4]. MT and even multimodal CT cannot be performed in most primary stroke centers (PSCs) due to equipment and emergency management method restrictions. Patients who have suspected LVO need to be transferred to a CSC for further evaluation and possible MT. For patients seen within an onset time <4.5 h, PSCs can perform IVT prior to transporting patients to the CSC, a method referred to as drip and ship (DS). Patients beyond the time window or in whom IVT is contraindicated, may be transferred to CSC directly (non-drip and ship, non-DS). Our CSC retrospectively summarized cases from November 2020 to May 2022 to compare three treatment strategies' clinical outcomes and explore the efficacy and safety of DS.

## 2 Methods

### 2.1 Study design

**Fig. 1:**
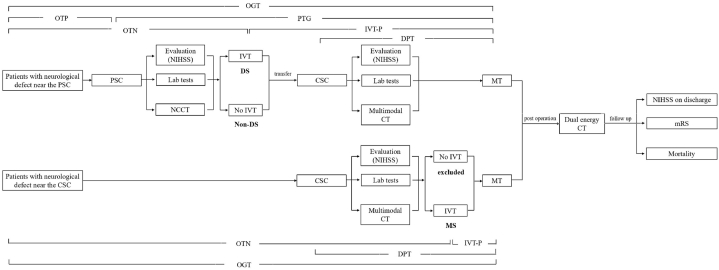
The transfer and treatment process of three transfer model. DS: drip and ship; non-DS: non drip and ship; MS: mother ship; PSC: primary stroke center; CSC: comprehensive stroke center; NCCT: non contrast CT; IVT: intravenous thrombolysis; MT: mechanical thrombectomy; SICH: symptomatic intracranial hemorrhage; NIHSS: National Institutes of Health Stroke Scale; mRS: modified Rankin scale; DPT: door to puncture time; OGT: onset to groin puncture time; OTN: onset to needle time; IVT-P: intravenous thrombolysis to puncture time; OTP: onset-to-PSC time; PTG: PSC-to-groin-puncture time.

We retrospectively reviewed the Beijing Chaoyang hospital databases of picture archiving and communication systems and the clinical information system for patients who presented to our institution with AIS for MT between November 2020 and May 2022. (Patients who were admitted directly to Beijing Chaoyang hospital for MT only were excluded). The approval for this study was obtained from the institutional ethics committee. According to whether the patient transferred from a PSC and whether they received IVT before MT, they were divided into three groups: 1) patients who received IVT in a PSC and then transferred to the CSC for MT (DS); 2) patients who were directly transferred to CSC for MT after preliminary evaluation in a PSC without IVT (non-DS); and 3) patients who were admitted to CSC for bridging thrombolysis (MS). Inclusion criteria were: 1) age ≥18; 2) demonstrated presence of LVO by multimodal CT; 3) ischemic core volume of less than 70 ml, a ratio of volume of ischemic tissue to ischemic core of 1.8 or more, and an absolute volume of penumbra of 15 ml or more; 4) no hemorrhagic stroke observed in CT scan; and 5) good baseline functional status (mRS ≤2). There are two PSCs in the health alliance, and both hospitals are about 1 h drive away from our CSC (about 55 km). We evaluated and compared the three groups in time course including the time from onset to the beginning of IVT (onset to needle time, OTN), the time from onset to the beginning of MT (onset to groin puncture time, OGT), the time from onset to PSC admission (onset to PSC time, OTP), the time from leaving PSC to the beginning of MT (PSC to groin puncture time, PTG), the time from CSC admission to the beginning of MT (door to puncture time, DPT), the time from beginning of IVT to the beginning of MT (IVT to groin puncture time, IVT-P), as well as ischemic core volume, ischemic penumbra volume, postoperative complications including symptomatic intracranial hemorrhage (sICH) and malignant edema based on multimodal CT, postoperative perfusion status grading (modified thrombolysis in cerebral infarction, mTICI), function outcomes such as admission and discharge National Institutes of Health Stroke Scale (NIHSS) scores, 90-day and long-term modified Rankin scale (mRS) score (>1 year) , 90-day mortality. Data were collected during 12–30 months follow-up. The transfer and treatment process are shown in Fig. 1.

### 2.2 Therapeutic processes

DS patients: Patients went to the PSC within 4.5 h of AIS onset. The PSC emergency doctor and neurologist evaluated and recommended standard IVT therapy based on a non-contrast CT (NCCT) scan, clinical symptoms, and guidelines. If the neurological deficiency was not resolved with the NIHSS score ≥6 or worsened during the observation process with the increase in the NIHSS score, the transfer mechanism to our hospital began, and the relevant information and treatment process conveyed to CSC doctors at the same time. During transport, the patient was monitored closely for any changes in symptoms. Once patients arrived at the CSC, the green channel integrated management strategy began immediately [5]. Multimodal CT was performed as soon as possible to evaluate cerebral vessel lesions. Patients who met the indications for MT were immediately bridged for MT.

non-DS patients: Patients arrived at the PSC beyond 4.5 h of AIS onset with the NIHSS ≥6. The transfer mechanism to the CSC began immediately. The admission process in the CSC after transfer was the same as the DS group.

MS patients: Patients were directly admitted to the CSC emergency within 4.5 h of AIS onset. Once patients arrived at CSC, the green channel integrated management strategy began immediately. Multimodal CT was performed immediately to evaluate cerebral vessel lesions. Patients who met the indications for BT immediately received BT therapy.

### 2.3 Evaluation before operation

All patients were assessed using the NIHSS score on admission. The patient's perfusion of brain tissue and intracranial blood vessels was evaluated through multimodal CT, including NCCT, CT angiography (CTA), and CT perfusion (CTP). Images were acquired using the GE Revolution Frontier CT (GE Healthcare, Waukesha, Wisconsin, USA) with 64 slices. In CTP, the ischemic core was defined as the region of reduced CBF (<30% of that in normal tissue) and the ischemic penumbra was defined as the tissue for which there was delayed arrival of an injected tracer agent (Tmax >6 s). The volume of ischemic core and ischemic penumbra were calculated with the use of the eStroke software (Neusoft, Shenyang, China).

### 2.4 Evaluation after operation

All patients were re-assessed with an NIHSS score on discharge. Safety outcomes were assessed by dual-energy CT. Malignant edema was defined as a syndrome of clinical worsening with imaging evidence of brain swelling (or requirement for decompressive hemicraniectomy or death) [6]. SICH was defined as any apparently extravascular blood in the brain or within the cranium that was associated with clinical deterioration, as defined by an increase of 4 points or more in the score on the NIHSS, or that led to death and that was identified as the predominant cause of the neurologic deterioration [7]. Two independent experienced neurosurgeons who were blinded to clinical outcomes rated recanalization success based on final angiograms according to mTICI score and functional independence according to mRS. Successful recanalization was defined as mTICI 2b-3 [8]. Good functional outcome was defined as mRS 0–2.

### 2.5 Statistical analysis

All analyses were performed using SPSS Statistics software, version 26.0 (IBM Corporation, New York, NY). Continuous variables are presented as mean (SD) or median values and interquartile range (IQR). Categorical variables are reported as frequency. Continuous variables such as age, NIHSS score, time course, and imaging characteristics were analyzed with an independent sample *t*-test, one-way ANOVA with Bonferroni post-hoc test, or Mann-Whitney test. Rank variables such as mTICI scores and postoperative mRS scores were analyzed with a rank sum test. Categorical variables such as past history, hemorrhage transformation, malignant edema and 90-day mortality were analyzed with a Chi-square test. Outcomes with *P* values < 0.05 were considered significant.

## 3 Results

**Fig. 2:**
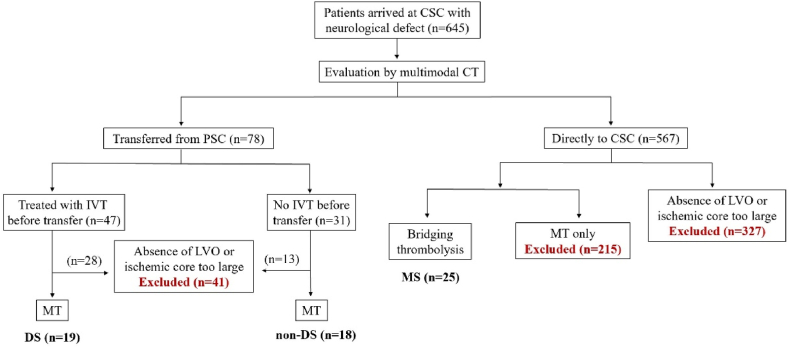
Schematic of three acute ischemic stroke triage decision model. DS: drip and ship; non-DS: non drip and ship; MS: mother ship; PSC: primary stroke center; CSC: comprehensive stroke center; LVO: large vessels occlusion; IVT: intravenous thrombolysis; MT: mechanical thrombectomy.

From November 2020 to May 2022, 318 patients were treated in our hospital for MT, of which 78 patients were transferred from a PSC to our hospital for evaluation, and 240 patients were directly treated in our hospital. Of the 78 patients transferred, 41 were excluded, 28 that received IVT before transport and 13 that did not. Of the 28 patients that received IVT, 26 were excluded because they had no LVO, and 2 were excluded because of the large volume of core infarction. Of the 13 patients who did not receive IVT, 7 were excluded because they had no LVO, and 6 were excluded due to large volume of core infarction. For the 240 patients treated directly at our hospital, 25 patients that underwent BT were included and 215 patients treated with MT alone were excluded. Thus, there are final dataset included 62 patients: 19 DS, 18 non-DS and 25 MS (Fig. 2). Details of the three groups are shown in Table 1. Baseline characteristics such as age, gender, past history, and preoperative NIHSS scores did not differ among the three groups.

**Table 1:** Baseline and clinical characteristics, safety outcomes and time course of patients in drip-and-ship, non-drip-and-ship, and mothership.

	DS (n = 19)	non-DS (n = 18)	MS (n = 25)	*P*-value
Age, mean (SD), y	70.1 (9.6)	67.5 (11.4)	63.2 (14.3)	0.312
Male, No. (%)	15 (78.9)	12 (66.7)	19 (76)	0.714
Hypertension, No. (%)	12 (75)	13 (75)	17 (70.6)	0.889
Diabetes, No. (%)	9 (47.4)	7 (38.9)	9 (36)	0.761
CHD, No. (%)	6 (31.6)	7 (38.9)	6 (24)	0.575
Hyperlipidemia, No. (%)	3 (15.8)	4 (22.2)	4 (16)	0.839
Atrial fibrillation, No. (%)	4 (21.1)	6 (33.3)	5 (20)	0.569
Stroke, No. (%)	8 (42.1)	5 (27.8)	6 (24)	0.474
Smoking, No. (%)	9 (47.4)	5 (27.8)	13 (52)	0.291
Drinking, No. (%)	8 (42.1)	7 (38.9)	10 (40)	1
Admission NIHSS, mean (SD)	13.12 (8.9)	13.51 (7.7)	11.42 (4.8)	0.692
Discharge NIHSS, mean (SD)	7.23 (8.3)	11.79 (12.33)	11.46 (11.35)	0.514
mTICI, No. (%)				0.157
0-2a	0	3 (16.7)	4 (16)	
2b-3	19 (100)	15 (83.3)	21 (84)	
sICH, No. (%)	2 (10.5)	1 (5.6)	3 (12)	0.872
Malignant edema, No. (%)	3 (15.8)	3 (16.7)	4 (16)	1
90-day mortality, No. (%)	1 (5.3)	2 (11.1)	0	0.185
90-day mRS, No. (%)				0.944
0-2	6 (31.6)	7 (38.8)	9 (36)	
3-6	13 (68.4)	11 (61.2)	16 (64)	
Time course (min)				
DPT, mean (SD)	109.2 (36.3)	111.9 (36.5)	126.1 (44.2)	0.591
OGT, median (IQR)	395 (326–500)	441.5 (398.5–635.25)	244 (159–284)	<0.001
OTN, mean (SD)	111.8 (45.3)		151.2 (47.7)	0.041
IVT-P, median (IQR)	278 (186–390.75)		56 (39.5–102)	<0.001
OTP, median (IQR)	90 (82–116)	463 (335–787)		<0.001
PTG, median (IQR)	277 (204–364)	162 (105–240)		0.002
Volume in CTP (ml), median (IQR)				
Ischemic core	10.94 (0.52–24.65)	11.09 (5.11–34.93)	9.69 (3.5–55.35)	0.662
Ischemic penumbra	144.13 (118.81–218.47)	179.09 (90.59–216.43)	139.48 (122.79–218.16)	0.957

DS: drip and ship; non-DS: non drip and ship; MS: mother ship; CHD: coronary heart disease; NIHSS: National Institutes of Health Stroke Scale; mTICI: modified Thrombolysis in Cerebral Infarction; sICH: symptomatic intracranial hemorrhage; mRS: modified Rankin scale; DPT: door to puncture time; OGT: onset to groin puncture time; OTN: onset to needle time; IVT-P: intravenous thrombolysis to puncture time; OTP: onset-to-PSC time; PTG: PSC-to-groin-puncture time; PSC: primary stroke center; CTP: computed tomography perfusion.

In terms of time course, DPT did not differ among the three groups (*P* = 0.591). OGT did vary among groups (*P* < 0.001), with the DS group being significantly longer than the MS group (DS median, 395 min; IQR, 326–500 min versus MS median, 244 min; IQR, 159–284 min; *P* < 0.001). The DS group had a shorter OTP than the non-DS group (DS median, 90 min; IQR, 82–116 min versus non-DS median, 463 min; IQR, 335–787 min; *P* < 0.001), but a longer PTG than the non-DS group (DS median, 277 min; IQR, 204–364 min versus non-DS median, 162 min; IQR, 105–240 min; *P* = 0.002). OTN was significantly longer in the MS group (mean, 151.2 min; SD 47.7 min) than in the DS group (mean 111.8 min; SD 45.3 min) (*P* = 0.041). IVT-P was shorter in the MS group compared to the DS group (MS median, 56 min; IQR, 39.5–102 min versus DS median, 278 min; IQR, 186–390.75 min, *P* < 0.001).

In terms of multimodal CT features, the three groups did not differ statistically in volume of ischemic core (DS median, 10.94 ml; IQR, 0.52–24.65 ml versus non-DS median, 11.09 ml; IQR, 5.11–34.93 ml versus MS median, 9.69 ml; IQR, 3.5–55.35 ml; *P* = 0.662) or ischemic penumbra (DS median, 114.13 ml; IQR, 118.81–218.47 ml versus non-DS median, 179.09 ml; IQR, 90.59–216.43 ml versus MS median, 139.48 ml; IQR, 122.79–218.16 ml; *P* = 0.957).

No significant differences among the three groups were observed in post-operative variables measured. All three groups obtained high rates of successful recanalization (DS: 100%; non-DS: 83.3%; MS: 84%; *P* = 0.157). Postoperative sICH did not differ among the three groups (DS: 2/19; non-DS: 1/18; MS: 3/25; *P* = 0.872). Postoperative malignant cerebral edema did not differ among the three groups (DS: 3/19; non-DS: 3/18; MS: 4/25; *P* = 1). The 90-day mortality did not differ among groups (DS: 1/19; non-DS: 2/18; MS: 0/25; *P* = 0.185). The rates of good outcome in 90-day among DS (31.6%), non-DS (38.8%) and MS (36%) groups did not significantly differ (*P* = 0.944). The final time of long-term follow-up among patients was 17.3 ± 4.9 months due to the different follow-up in three groups. The follow-up rate was about 80.6%. Finally, there was no difference in the rates of good outcome in long-term follow-up among DS (8/17, 47.1%), non-DS (5/14, 35.7%) and MS (8/19, 42.1%) group (*P* = 0.816).

**Fig. 3:**
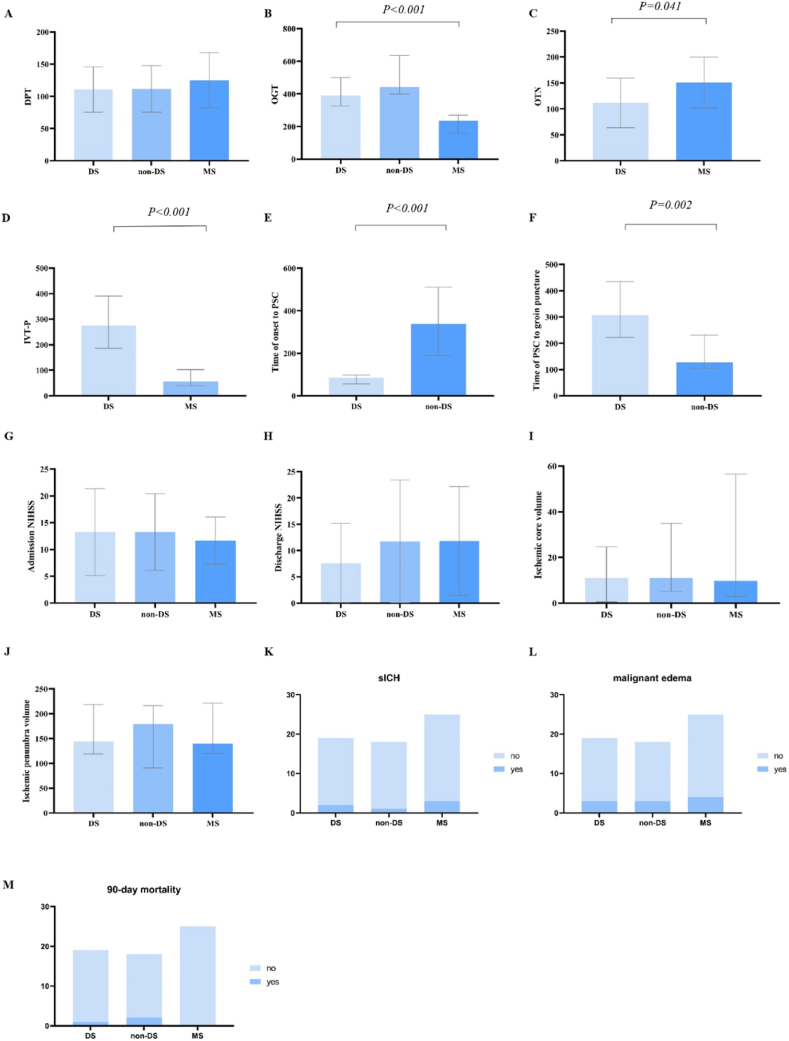
Results of between groups comparison with Bonferroni correction in DPT (A), OGT (B), admission and discharge NIHSS (G, H), ischemic core volume (I), ischemic penumbra volume (J), sICH (K), malignant edema (L), and 90-day mortality (M). And time course between groups such as OTN (C), IVT-P (D), OTP (E), and PTG (F) compared by unpaired *t*-test and Mann-Whitney test.

**Fig. 4:**
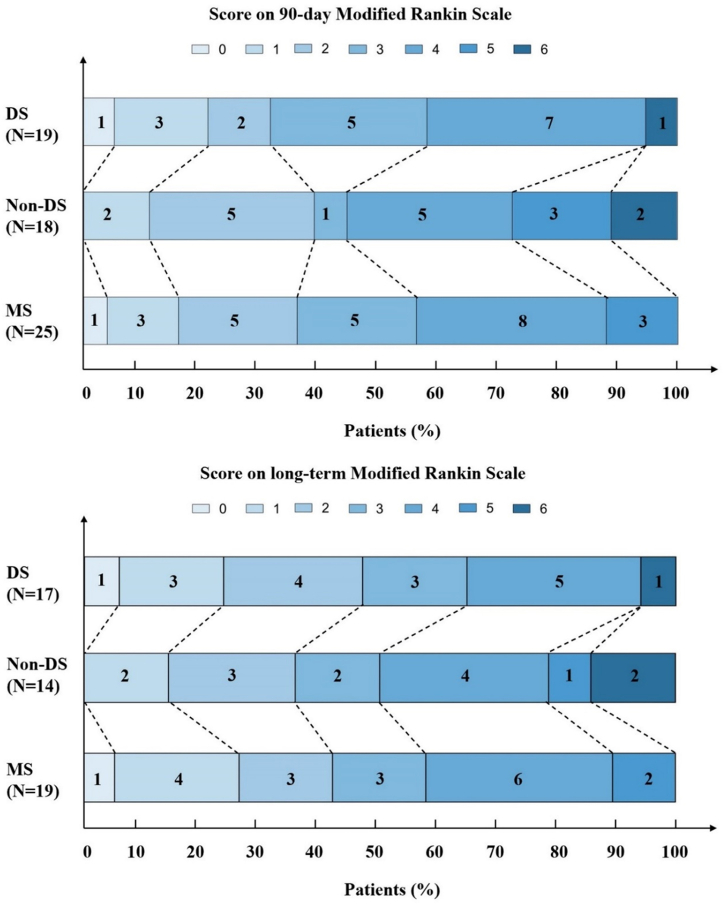
Distribution of the mRS score at 90 days and long term in patients treated with DS paradigm, non-DS paradigm, and MS paradigm. DS: drip and ship; non-DS: non drip and ship; MS: mother ship.

The results of analyses are shown in Fig. 3. Fig. 4 shows clinical outcomes of patients between three groups.

## 4 Discussion

In this study, we explored the safety and efficacy of thrombolysis in the process of transport and treatment by comparing the two transport methods of DS and non-DS. There was no significant difference in prognosis between two groups. Currently, most PSCs cannot perform multimodal CT evaluation in China. Patients who meet the conditions for thrombolysis, are transferred to the CSC for imaging evaluation and MT after receiving IVT. Transport did not appear to increase the risk of post-thrombolytic cerebral hemorrhage, postoperative malignant cerebral edema, or death, as all groups were similar for these outcomes. In the non-DS group, most patients did not receive thrombolysis because the thrombolytic time window had passed, so the OTP was significantly longer in these patients than in the DS group (non-DS median, 463 min; IQR, 335–787 min versus DS median, 90 min; IQR, 82–116 min; *P* < 0.001). The distance from the PSCs to our hospital is about 55 km, and the actual transit time is about 78.3 min. The median PTG of the DS group being 115 min longer than that of the non-DS group and leading to no statistical difference in OGT between two groups (DS median, 395 min; IQR, 326–500 min versus non-DS median, 441.5 min; IQR, 398.5–635.25 min; *P* > 0.05). The main reason is that the patients did not start the referral system immediately after IVT in the PSC but instead continued to be observed during IVT. The referral program was only started when it was determined that patient conditioned unchanged or worsened after thrombolysis. This delayed the endovascular therapy for patients. Once IVT is given, the transport mechanism to CSC should be activated immediately to accelerate the cerebral reperfusion and improve the prognosis of patients. The data of our study was collected from Beijing Chaoyang hospital of Capital Medical University and medical consortium. The statistical results showed that the OGT of our study was longer than other studies [[9], [10], [11], [12]]. Our center has established an integrated health care system with PSC, and medical conditions in capital of China is developed relatively. In fact, the OGT may be longer in most areas of China. Therefore, optimizing the admission and treatment process of the PSC and establishing a perfect transfer system is necessary for AIS.

Previous data from cohorts such as STRATIS registry show that patients in the MS group present a higher chance of functional independence [13]. Adams et al. [14] provide an exception, having found similar outcomes after thrombectomy in the MS and DS groups. According to our data, the DS group had a longer OGT than the MS group (DS median, 395 min; IQR, 326–500 min versus MS median, 244 min; IQR, 159–284 min; *P* < 0.001), but this did not coincide with any significant difference in the rates of sICH, malignant cerebral edema, or 90-day mortality. Studies have shown that the most important factor for obtaining good functional outcomes is rapid cerebral reperfusion and the shorter the OGT, the better the prognosis [15]. In our study, the OGT in the DS group was longer, but the thrombectomy effect was enhanced without increasing the complication rate; this may reflect benefits of thrombolysis before transport. Previously published studies showed shorter OTN in MS group [12,16,17]. In comparison, the DS group had shorter OTN than the MS group (DS, mean,111.8 min; SD 45.3 min versus MS, mean, 151.2 min; SD 47.7 min; *P* = 0.041), which we attribute to the smaller scope of services offered by PSCs and smooth traffic in remote areas. In addition, patients in the DS group received IVT earlier than the MS group, which is also an important factor in patient prognosis. Finally, although the two groups did not significantly differ in DPT, the DS group tended to have a shorter DPT than the MS group (DS, mean, 109.2 min; SD 36.3 min versus MS, mean, 126.1 min; SD 44.2 min, *P* = 0.346). The early warning by the PSC before transfer may lead to the shorter DPT in the DS group and in timely treatment of patients transferred to CSC; this is consistent with results of previous studies [17,18].

Early IVT can be more effective for distal vascular occlusion caused by small thrombi than for LVO [19]. A meta-analysis of 13 studies showed that only 11% of patients with LVO who received IVT achieved recanalization [20]. In our data, all patients in the DS group achieved successful recanalization, whereas three patients in the non-DS group and four patients in the MS group did not. In addition, 26 patients with no LVO detected by multimodal CT after transport to our hospital may have benefitted from thrombolysis before transport. After completion of the cerebrovascular imaging examination in the CSC, only two patients in the DS group had an ischemic core volume exceeding 70 ml. Since the PSC did not complete the cerebral vascular imaging evaluation, we cannot obtain an accurate measure of cerebral perfusion before transfer; we are thus unable to assess the effect of thrombolytic therapy in patients prior to transport. Since thrombolysis has limitation on the recanalization of LVO, we speculate while thrombolysis likely failed to achieve the recanalization of vessels in patients with LVO in the DS group, it may have led to the establishment of collateral circulation [21]. This response delays the progression of the ischemic core without increasing the probability of sICH, and is thus beneficial for brain tissue reperfusion and postoperative neurological recovery.

With the publication of the DAWN and DEFUSE-3 studies, imaging-based physiological information may shift the treatment paradigm from a rigid time-based model to a more flexible and individualized, tissue-based approach, increasing the proportion of patients considered appropriate for treatment. Multimodal CT includes NCCT, CTA, and CTP. It can achieve rapid and accurate assessment of LVO, collateral circulation, volume of ischemic penumbra and volume of ischemic core [22]. However, in China and other developing countries, most PSCs can only preliminarily assess patients using NCCT to assess whether bleeding lesions and severe cerebral infarction are present; such facilities cannot assess the presence of LVO and abnormal cerebral perfusion accurately. The first multimodal CT assessment can only be completed after transported to the CSC. NCCT is widely used in the evaluation of neurocritical care as it has the benefits of being fast and effective. It can provide information for the identification of hemorrhage and ischemic lesions quickly, but it has limitations in detection of early ischemic lesions [23]. The Alberta Stroke Program Early CT score (ASPECTS) is an important method to quantify early ischemic changes based on NCCT imaging, and it is widely used in clinical practice for its simplicity and effectiveness. However, clinical decisions can differ from one clinician to the other [24]. In addition, the prediction of the prognosis of AIS patients based on the ASPECTS is controversial. The results of the MR CLEAN study showed that a higher ASPECTS predicts good prognosis of patients [25]. However, the SWIFT PRIME and ESCAPE studies found no significant difference in prognosis, intracranial hemorrhage and mortality between patients with ASPECTS 4–6 and those with ASPECTS 7–10 [26,27]. Insensitivity to the diagnosis of early ischemic lesions limits the role of NCCT in thrombectomy decision-making. In the present study, multimodal CT assisted us to screen patients who were transferred from a PSC to our hospital. A total of 28 patients without LVO or with large volume of ischemic core were excluded from our study. Among the enrolled patients, multimodal CT also helped us to identify the location of the LVO, the status of collateral circulation and the perfusion of brain tissue, and provided guidance and help for MT methods. If PSCs are able to perform multimodal CT evaluation, the vascular occlusion and cerebral tissue perfusion can be evaluated and screened at the first visit of the patient, thereby reducing the potential safety hazards caused by unnecessary transport, improving economic benefits, and achieving more precise treatment.

A limitation of our study is the small size sample and that it included cases in the 18 months only. In future, we will include more cooperative medical institutions, and prospectively collect a larger sample to verify conclusions. Another limitation is the restrictions of equipment in PSCs. AIS patients cannot receive multimodal CT in the PSC, and thus we cannot ascertain whether thrombolysis achieves recanalization of occluded vessels, or whether the thrombus dissolves and migrates. Finally, this study was a retrospective study, most patients in the non-DS group did not receive IVT therapy during the enrollment due to exceeding the thrombolytic time window. Therefore, time from onset to the first visit was longer than the DS group, which may lead to a trend of worse prognosis in the non-DS group. In future, prospective studies could be conducted with inclusion of patients within the thrombolytic time window during enrollment of non-DS group to eliminate bias.

In conclusion, the DS method is safe and effective, with no increased risk of postoperative hemorrhage transformation, malignant edema or death compared to non-DS and MS methods. The study provides a reference for the selection of transport modes for AIS patients.

## Author contribution statement

1 - Fucheng Jiang; Wenpeng Yin; He Liu: Conceived and designed the experiments;

2 - Fucheng Jiang; Wenpeng Yin; He Liu: Performed the experiments;

3 - Fucheng Jiang; Wenpeng Yin; He Liu: Analyzed and interpreted the data;

4 - Jianwen Jia; Hongliang Zhong; Hongchao Yang; Jvmei Huang; Yang Wang; Yunpeng Liu; He Liu: Contributed reagents, materials, analysis tools or data;

5 - Fucheng Jiang; Wenpeng Yin; He Liu: Wrote the paper.

## Statement of ethics

This retrospective study was approved by the ethics committee of Beijing Chaoyang Hospital (2022-D-218) according to the principles expressed in the Declaration of Helsinki. Written informed consent was obtained from individual participants.

## Funding sources

This research did not receive any special grant from funding agencies in the public, commercial, or not-for-profits sectors.

## Data availability statement

Data will be made available on request.

## Declaration of competing interest

The authors declare that they have no known competing financial interests or personal relationships that could have appeared to influence the work reported in this paper.
